# Brain Abscess Likely Secondary to Infective Endocarditis Mimicking High-Grade Glioma in a Patient with Pyelonephritis

**DOI:** 10.7759/cureus.110601

**Published:** 2026-06-10

**Authors:** Taytem M Rath, Shakshi Patel, Dilja Mary David, Sethu M Muralidharan, Shadab H Ahmed

**Affiliations:** 1 Internal Medicine, American University of the Caribbean School of Medicine, East Meadow, USA; 2 Internal Medicine, Nassau University Medical Center, East Meadow, USA; 3 Infectious Disease, Nassau University Medical Center, East Meadow, USA

**Keywords:** acute pyelonephritis, brain abscess, delayed diagnosis, high-grade glioma, infective endocarditis, intracranial abscess, mimicry, mri brain, ring-enhancing, septic emboli

## Abstract

Brain abscesses are serious central nervous system infections that often present with nonspecific infectious and neurologic symptoms and may mimic intracranial neoplasms on imaging. They can develop rapidly or insidiously, and diagnosis is often delayed when symptoms overlap with concurrent systemic infections. We report the case of a 66-year-old woman admitted for pyelonephritis who initially improved with intravenous antibiotics but later developed persistent fever, worsening occipital headache, and acute neurologic deterioration. Initial imaging demonstrated an irregular ring-enhancing lesion suggestive of high-grade glioma; however, intraoperative findings and histopathology confirmed a brain abscess. Gram stain revealed anaerobic gram-positive cocci in pairs, but cultures showed no growth. Further evaluation identified a mitral valve vegetation highly suggestive of infective endocarditis (IE), representing the most likely source of septic embolization; however, a definitive microbiologic link could not be established because blood cultures remained negative and the causative organism was not identified. This case highlights the diagnostic difficulty of brain abscess when it mimics an intracranial neoplasm and occurs alongside systemic infection. Importantly, it also underscores the need to maintain a broad differential diagnosis when clinical status worsens despite appropriate therapy and to consider IE as an occult source of septic emboli in patients with unexplained neurologic decline and concurrent infection.

## Introduction

Brain abscess is a rare but life-threatening intracranial suppurative collection commonly affecting the cerebral hemispheres, with possible fungal, mycobacterial, parasitic, and bacterial sources [1]. It may arise through contiguous spread from nearby infections, hematogenous dissemination, and through direct inoculation, with some cases remaining cryptogenic [1,2]. Hematogenous spread is frequently associated with cardiac sources, particularly infective endocarditis (IE), which can result in septic embolization to the brain [1].

Importantly, a subset of IE cases is culture-negative due to prior antimicrobial exposure, fastidious organisms, or atypical pathogens, which may significantly delay diagnosis and complicate microbiologic confirmation despite strong clinical and echocardiographic evidence of infection. In a large multicenter cohort of 1,345 patients with IE, 340 patients developed neurologic complications, with 2 cases (1%) representing brain abscess. Septic embolization, as a result of IE, more commonly manifests as ischemic infarction, hemorrhage, or clinically silent microabscesses rather than the formation of a mature intracranial abscess [3]. Despite advances in antimicrobial and neurosurgical techniques, brain abscesses continue to carry substantial neurologic morbidity and mortality of approximately 21% at one year and 16% within 2-5 years [4].

Patients most commonly present with neurologic manifestations related to mass effect, increased intracranial pressure, or focal cortical involvement, dependent on abscess size, location, and associated cerebral edema. Typical presenting symptoms include worsening headaches, fever, changes in mentation or vision, seizures, and focal deficits, such as weakness and speech or sensory disturbances [1]. Imaging typically shows ring-enhancing lesions with surrounding vasogenic edema, but these findings may mimic other intracranial pathologies such as neoplasms, metastases, or demyelinating processes, posing a diagnostic challenge, especially with toxoplasmosis and lymphomas [1,5]. Diffusion-weighted imaging (DWI) with apparent diffusion coefficient (ADC) mapping provides important diagnostic value, as pyogenic brain abscesses characteristically demonstrate marked central restricted diffusion due to viscous purulent content, helping distinguish them from high-grade gliomas, which more commonly demonstrate heterogeneous or peripheral diffusion restriction patterns [5].

Although neurologic symptoms are the most frequent manifestations, atypical or misleading presentations can often delay diagnosis, especially when physical exam and laboratory results are nonspecific [6]. We present a case of a brain abscess initially suspected to be high-grade glioma in a patient undergoing treatment for pyelonephritis. This abscess was ultimately found to be likely secondary to IE, emphasizing the importance of reassessing the differential diagnosis when patients deteriorate despite appropriate antimicrobial therapy.

## Case presentation

A 66-year-old woman with a history of hypertension presented with one week of abdominal pain radiating to the right flank, accompanied by nausea, diarrhea, dysuria, and generalized weakness. Her family reported a consistently normal level of consciousness prior to admission.

On examination, she was afebrile but tachycardic (110-115 bpm), with diffuse abdominal tenderness most pronounced in the right upper quadrant. Initial laboratory evaluation revealed significant leukocytosis of 25.67 x 10^3^/μL and hyperglycemia of 445 mg/dL. Further evaluation demonstrated previously unrecognized type 2 diabetes mellitus with a hemoglobin A1c of 13.7%, indicating chronic poor glycemic control. A clean-catch urinalysis revealed pyuria, hematuria, many bacteria, and yeast, while urine culture grew pan-sensitive *Escherichia coli*. Computed tomography (CT) findings were consistent with pyelonephritis, and the patient received a one-time dose of piperacillin/tazobactam and fluconazole, after which she was admitted for management with intravenous (IV) ceftriaxone.

Initially, the patient showed clinical improvement, with stabilization of vital signs (temperature 36.5-37.1℃ (97.7-98.8°F), heart rate 78-89 bpm, blood pressure 120-140/70-85 mmHg, and oxygen saturation ≥95% on room air), improvement in leukocytosis to 10.23 x 10^3^/μL, and stable mental status. However, by hospital day 4, she developed recurrent fevers with a maximum temperature (Tmax) of 38.7℃ (101.6°F) despite antibiotic therapy. Repeat laboratory evaluation at that time demonstrated the following (Table 1).

**Table 1:** Repeat laboratory findings on day 4

	Result	Reference range
Hematology		
White blood cell count	10.61 x 10^3^/μL	4.5-11.0 x 10^3^/μL
Hemoglobin	7.7 g/dL	12.0-15.5 g/dL
Hematocrit	23.80%	36-44%
Platelet count	533 x 10^3^/μL	150-450 x 10^3^/μL
Liver function tests		
Aspartate aminotransferase	19 U/L	8-48 U/L
Alanine aminotransferase	20 U/L	7-45 U/L
Alkaline phosphatase	184 U/L	44-147 U/L
Total bilirubin	0.7 mg/dL	0.2-1.3 mg/dL
Renal function tests		
Blood urea nitrogen	12 mg/dL	7-20 mg/dL
Serum creatinine	0.8 mg/dL	0.5-1.1 mg/dL
Creatinine clearance	111 mL/min	72-110 mL/min

Infectious disease was consulted, and her antibiotic regimen was adjusted to IV cefepime. Despite these measures, fevers persisted on hospital day 5 with a Tmax of 39.2℃ (102.6°F), resulting in initiation of IV metronidazole with subsequent stabilization of temperatures. During this time, she developed a severe occipital headache requiring analgesics. Repeated blood cultures remained negative, with repeat urinalysis demonstrating sterile pyuria and trace blood.

On hospital day 11, she became acutely unresponsive and subsequently demonstrated altered mental status with a Glasgow Coma Scale score of 11. She concurrently developed recurrent high-grade fever (Tmax 39.1℃ (102.3°F)), prompting targeted escalation of antimicrobial therapy to IV meropenem and linezolid, after which febrile episodes were controlled as both agents demonstrate significant penetration of the blood-brain barrier.

Given the complexity of the clinical course, a summarized timeline of major diagnostic and therapeutic events is provided in Table 2.

**Table 2:** Clinical timeline of major hospital events TTE: transthoracic echocardiography, TEE: transesophageal echocardiography.

Hospital day	Clinical event
0	Admission with pyelonephritis; IV ceftriaxone initiated
1-3	Clinical improvement with declining leukocytosis and stable mental status
4	Recurrent fevers despite therapy; transition to cefepime
5	Persistent fever and severe occipital headache; metronidazole added
11	Acute neurologic decline with altered mental status; switch to meropenem and linezolid
11	CT head demonstrated a left parietal abnormality
11	MRI revealed a ring-enhancing lesion concerning for high-grade glioma
15	Craniotomy performed; brain abscess identified; switch to meropenem and vancomycin
16	TTE demonstrated moderate mitral and tricuspid regurgitation
19	TEE demonstrated mitral valve vegetation and a right-to-left intracardiac shunt
26	Discharged to complete 6 weeks of IV antibiotics and rehabilitation

Emergent non-contrast CT of the head revealed loss of gray-white differentiation in the left posterior parietal lobe, indicating ischemic infarct versus an underlying mass lesion (Figure 1). Magnetic resonance imaging (MRI) of the brain with and without IV gadolinium-based contrast revealed a 4.0 x 3.0 x 2.3 cm irregular ring-enhancing lesion with extensive perilesional edema (Figure 2). The lesion demonstrated restricted diffusion on DWI/ADC sequences, reflecting impaired movement of water molecules within the brain tissue. Given the irregular enhancement pattern and infiltrative appearance, these findings were initially interpreted as most consistent with a high-grade glioma.

**Figure 1:**
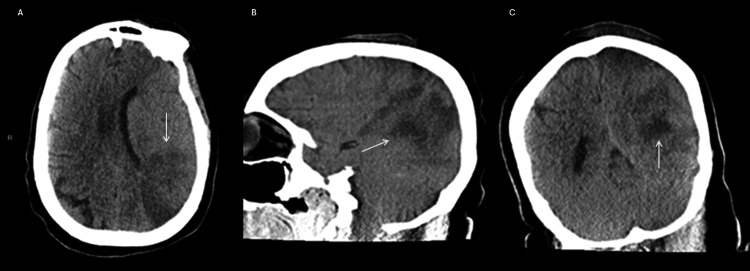
Noncontrast CT demonstrating left posterior parietal abnormalities (A-C) Axial, sagittal, and coronal CT of the brain without IV contrast demonstrating nonspecific loss of gray-white differentiation in the left posterior parietal lobe (white arrows).

**Figure 2:**
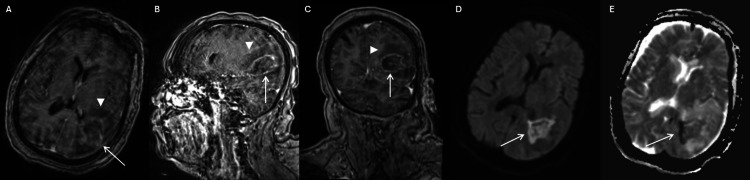
Brain MRI demonstrating left parietal ring-enhancing lesion (A-C) Axial, sagittal, and coronal T1-weighted MRI obtained following IV gadolinium-based contrast administration, demonstrating an irregular ring-enhancing lesion (white arrows) in the left posterior parietal lobe measuring 4.0 x 3.0 x 2.3 cm. Associated surrounding hypointense signal is consistent with extensive vasogenic edema (white arrowheads). (D) DWI trace sequence demonstrating marked restricted diffusion (white arrow) within the lesion. (E) ADC map demonstrating a corresponding low ADC signal (white arrow), further supporting restricted diffusion.

Neurological examination demonstrated minimal responsiveness, aphasia, right-sided neglect, tongue deviation to the right, and mild left-sided facial asymmetry. The patient was transferred to the surgical intensive care unit for close monitoring and neurosurgical evaluation, and was initiated on levetiracetam and dexamethasone.

During left-sided craniotomy, purulent material and necrotic tissue were identified intraoperatively, with no evidence of neoplasm. Histopathologic analysis of the material confirmed a brain abscess. Gram stain showed anaerobic gram-positive cocci in pairs, while cultures remained negative with no bacterial species identified, likely secondary to prolonged exposure to broad-spectrum antibiotics. Repeated blood cultures obtained throughout hospitalization were likewise negative, potentially reflecting prior antimicrobial therapy.

Subsequent workup to localize the source of infection included negative HIV and hepatitis serology, normal chest imaging, and no evidence of sinus, otic, or dental infection. Transthoracic echocardiography (TTE) showed moderate mitral and tricuspid regurgitation. Transesophageal echocardiography (TEE) identified a small (0.8 x 0.3 cm), pedunculated, mobile mitral valve vegetation and a small right-to-left atrial shunt. These findings were highly suggestive of IE and supported septic embolization as the most likely source of the patient’s cerebral abscess. However, a definitive microbiologic relationship could not be established because blood cultures remained negative, and no causative organism was isolated. Importantly, the endocarditis was considered independent of the patient’s pyelonephritis, with no evidence linking the urinary tract infection to the valvular infection or cerebral abscess formation.

Following surgical intervention, the patient completed a six-week course of IV meropenem and vancomycin. She subsequently underwent a prolonged inpatient physical medicine and rehabilitation stay and demonstrated gradual neurologic improvement over the course of therapy. She was later brought to the infectious disease clinic by wheelchair with family assistance. At follow-up, she was alert and oriented to person, place, and time, with grossly normal motor strength in all extremities and intact cranial nerve function. She was able to answer simple questions but remained unable to reliably respond to complex questions. There was no evidence of recurrent infection.

## Discussion

Brain abscess remains a rare condition with a global incidence of approximately 0.9 per 100,000 individuals and peak prevalence among adults aged 30-50 years [1,6]. Predisposing conditions play a critical role in abscess formation, including diabetes, immunosuppression, heart or pulmonary disease, and infections in adjacent or distant sites (e.g., sinusitis, mastoiditis, otitis media, dental infection, bacteremia) [7]. The most common causative organisms include *Streptococcus* species, *Staphylococcus aureus*, and anaerobic bacteria, with pathogen distribution often reflecting the source of infection [1]. In the present case, newly diagnosed uncontrolled diabetes mellitus, evidenced by a hemoglobin A1c of 13.7%, likely represented an additional risk factor because chronic hyperglycemia impairs host immune function and increases susceptibility to severe infection [1,8].

This case illustrates the diagnostic complexity of brain abscess, particularly when presentation is confounded by concurrent systemic infection. The patient’s initial diagnosis of pyelonephritis provided a plausible explanation for her presenting symptoms and subsequent fever, making recognition of evolving intracranial pathology more difficult. Because altered mental status and delirium frequently occur in older adults with infection, her neurologic decline could have easily been attributed to systemic illness alone. Additionally, stroke remained an important differential diagnosis given the acute neurologic deterioration and subsequent focal deficits. Persistent fever despite appropriate antimicrobial therapy, development of severe occipital headache, and eventual focal neurologic deficits ultimately prompted further investigation and led to diagnosis. 

Radiographic differentiation between brain abscess and high-grade glioma remains challenging because substantial overlap exists in their imaging characteristics. High-grade gliomas typically demonstrate irregular margins, heterogeneous enhancement, and infiltrative edema, whereas brain abscesses classically appear as smooth, thin-walled, ring-enhancing lesions with more homogenous internal contents and surrounding vasogenic edema [5]. However, these distinctions are not absolute, as abscesses may become irregular or multiloculated and closely mimic malignancy, as in this case [1,5]. Although restricted diffusion on DWI/ADC sequences is considered a characteristic feature of pyogenic abscess and was present in this patient, the lesion also demonstrated irregular ring enhancement, extensive edema, and an infiltrative appearance that raised concern for high-grade glioma. Consequently, the overall radiographic appearance was considered atypical for a classic brain abscess, and malignancy remained a leading preoperative consideration until surgical exploration established the diagnosis.

A key finding in this case was the identification of a mitral valve vegetation on TEE supporting probable IE as the most likely source of septic embolization, by Modified Duke criteria in the setting of culture-negative disease. However, this association should be interpreted cautiously. Blood cultures remained negative, and no causative organism was isolated from abscess cultures, precluding definitive microbiologic confirmation of infective endocarditis as the source of the cerebral abscess. Nevertheless, the absence of an alternative infectious source and the presence of valvular vegetation make a cardiac origin the most plausible explanation.

Overall, the patient met one major criterion (echocardiographic evidence of a mitral valve vegetation) and at least three minor criteria, including documented fever >38℃ (100.4°F), vascular phenomena in the form of cerebral septic embolization manifesting as a brain abscess, and the presence of predisposing cardiac pathology in the form of an intracardiac shunt. Although these findings approach the threshold for definitive IE under the Modified Duke criteria, the absence of microbiologic confirmation limits classification to probable IE [3].

The accompanying right-to-left atrial shunt may have contributed to the risk of cerebral embolization by facilitating passage of embolic material into the systemic circulation; however, its precise role cannot be determined and should be considered a potential rather than definitive pathogenic factor [4,8]. Once established in cerebral tissue, infection progresses through stages of cerebritis and capsule formation, accompanied by breakdown of the blood-brain barrier, release of inflammatory mediators, and development of vasogenic edema [1,8]. These processes contributed to increased intracranial pressure and focal neurologic deficits, which accounted for this patient’s acute clinical deterioration.

Management of brain abscess typically involves a combination of prolonged IV antibiotic therapy based on culture results and subsequent neurosurgical intervention dependent on abscess size, mass effect, or diagnostic uncertainty [7]. Initial supportive management with corticosteroids, anti-seizure medications, and early rehabilitation is integral to comprehensive care. Long-term neurologic sequelae, including persistent focal deficits, cognitive impairment, and post-infectious epilepsy, occur in approximately 70% of cases and highlight the importance of early recognition and prompt management [4].

## Conclusions

This case illustrates an unusual clinical scenario in which a radiographically misleading brain abscess emerged during hospitalization for pyelonephritis and was ultimately found to be associated with probable IE. It highlights the potential for serious intracranial pathology to be discovered during workup for unrelated conditions and reinforces the importance of maintaining a high index of suspicion when imaging reveals a ring-enhancing lesion. Evaluation for IE should be considered in patients with brain abscess, particularly when clinical deterioration occurs despite appropriate antimicrobial therapy.
